# Nomenclature as Applied to Dentistry

**Published:** 1893-11

**Authors:** 


### Editorial.

### NOMENCLATURE AS APPLIED TO DENTISTRY.

There seems to be an ever-increasing desire on the part of persons of restless energy to improve the English tongue, or at least to change it to their peculiar standard, and to multiply its already overburdened list of words. This in one sense may be said to be a laudable work, since all attempts in the direction of improvement may be regarded as of value. If nothing more be accomplished, these efforts arouse attention and in degree stimulate discovery.

The tendency of certain minds has been constantly in this direction for a period beyond the memory of the present generation, and yet the results are, thus far, of uncertain value even where adopted by a large body of people.

This feeling of unrest has, very naturally, communicated itself to technical nomenclature, and for a considerable period there have been periodical efforts made to improve the terms used. Dentistry being one of the youngest of the professions, this object has been more energetically pursued among its practitioners than elsewhere.

It could hardly be expected, therefore, that the recent Congress would pass without this subject being treated with more or less ability; and it has occasioned no surprise to find that several papers were read before the sections and that all of them were of value and exhibited much ingenuity. We have not had the pleasure of reading the official report in full, but the meagre abstract given leads to the conclusion that it will prove by far the ablest paper on nomenclature yet presented.

This being admitted, the question, after all, arises, What good can all this labor and intelligent thought accomplish? Beyond the mere fact that it reopens the entire subject as related to dentistry, and for a limited period may rouse minds to the consideration of the importance of better terms and more precise definitions in regard to places and conditions, it cannot advance, in the opinion of the writer, the matter one step in the direction proposed.

Language is a development, an evolution from simple roots, and, while its origin may be in a measure speculative, there are certain fixed bases of opinion, settled by philologists, that demonstrate quite conclusively that all forced attempts to modify or create a language in whole or in part must result in failure.

From the remote periods of picture-forming to the alphabets of the present day is an uncounted lapse of time, but it is not necessary to our purpose to follow the investigations and hypotheses of philologists in their attempts to explain the gradual development from pictures to root-forms and the rise and fall of languages. It is sufficient for the present object to know that these developments have been so slow that even in those languages capable of direct observation, as in the modern tongues, changes are made so imperceptibly that it requires the trained philologist to note the varying shades of difference from one generation to another.

It is very evident, however, that in many instances words acquire very slowly new meanings entirely independent of their origin, for, as Professor W. D. Whitney (*Encyc. Brit.*), says, "When once the name is applied, it belongs to that to which it is applied, and no longer to its relatives by etymology; its origin is neglected, and its form may be gradually changed beyond recognition or its meaning so far altered that comparison with the original shall seem a joke or an absurdity."

While it is true that a composite language, such as English, is made up mainly of adopted words, there necessarily are periods of intellectual progress in which there is a demand for clearer expression, and eventually this unexpressed thought will be clothed in a word adopted or coined to suit it; but it must be remembered that the thought-form preceded and had become part of the mentality of a considerable portion of a people before the new word found a birth and final assimilation in the language. The necessity for thought expression must precede word formation.

This being true, it is impossible to conceive of a series of words being formulated by an individual or a company of individuals, and that these should transfer themselves by any known process into the living thoughts of any large body of persons. The attempts made in this direction on a large scale have always proved a failure.

The slow adoption of single words demonstrates that changes in language structure must go through a variety of imperfect forms all tending towards more precise expression of the thought as yet but crudely expressed.

All words in the various languages have had an existence originally in mental pictures, and these constitute the spirit of the language. This is rarely caught in set definitions, and hence the student fails to acquire the idiomatic force of a tongue in proportion as he fails to absorb its spirit. It is simply the exemplification of the idea that thought precedes all word-building and that this thought must be sought for to comprehend the word.

If these propositions be true as briefly and imperfectly stated, it follows, as a natural sequence, that any attempt to build a technical structure for the adoption of a large body of persons, as the dental fraternity, must result in failure.

Improvements in expression are needed, and, it may be said, sadly needed. Dentistry has, in its short life, adopted many crude phrases and word-forms unscientific and indicative of untrained intelligence. They have been, however, representative of conditions and forms as they appeared in the earlier development of the profession, and it would seem better to bear with them as we bear with the child's prattle, until a better nomenclature can be slowly evolved. Learned treatises will not avail in this direction. The thought of the teachers of this epoch must find expression in more perfect forms, and as these slowly enter the minds of the pupils there will gradually be evolved more suitable words to replace the old, but this may require generations to accomplish.

